# Supplementary material for: The interactome of intact mitochondria by cross-linking mass spectrometry provides evidence for coexisting respiratory supercomplexes
Source: Mol Cell Proteomics. 2017 Dec 8;17(2):216–32. doi: 10.1074/mcp.RA117.000470 (PMC5795388; doi:10.1074/mcp.RA117.000470)
Supplement: Supplemental Data [file supp_RA117.000470_133922_0_supp_23978_fzffwf.zip › spectra_annotation/mito_spectra_annotation/343-1-9-1-4-1.pdf]

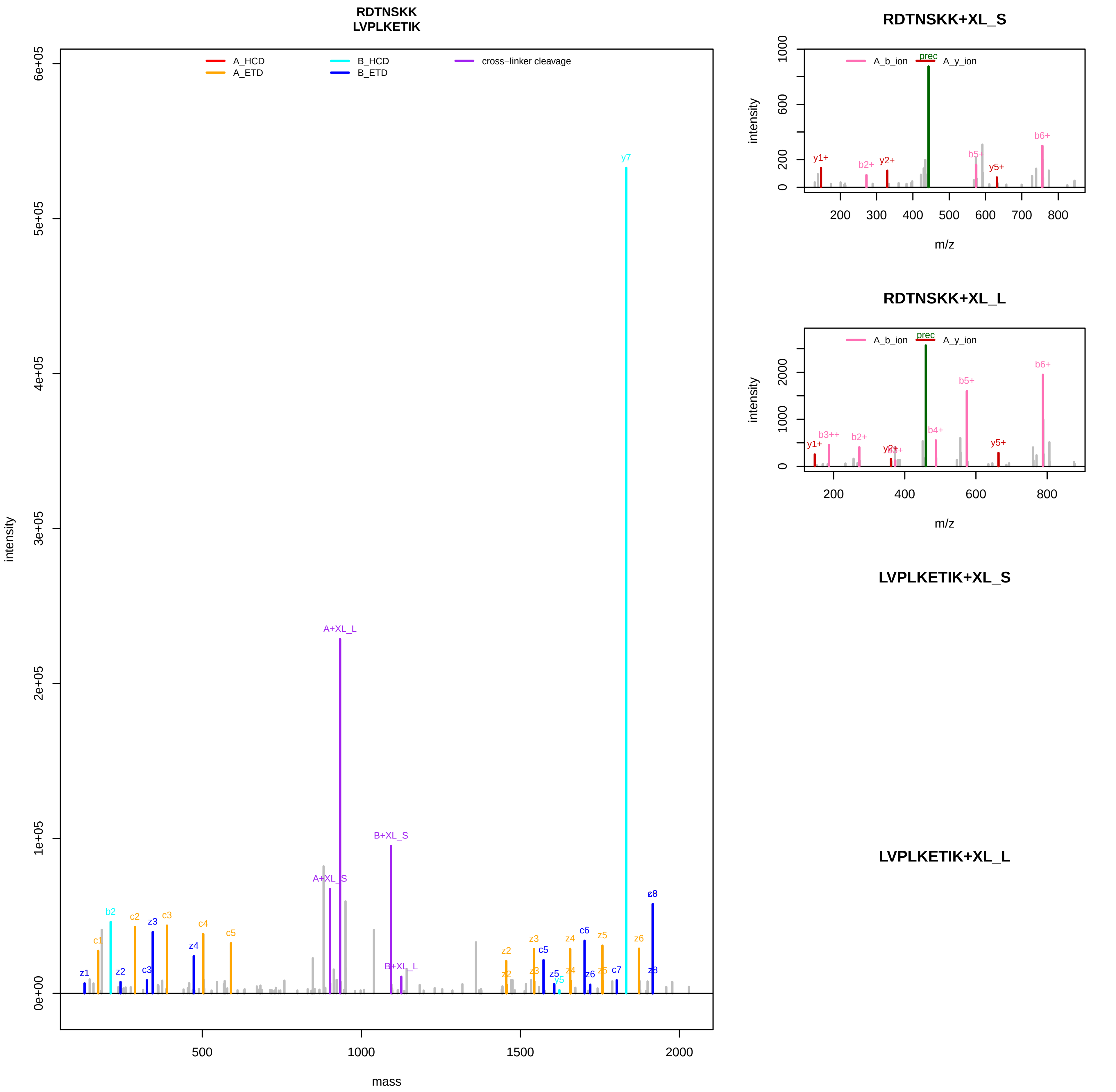
RDTNSKK
LVPLKETIK
A_HCD
A_ETD
B_HCD
B_ETD
cross−linker cleavage
y7
A+XL_L
B+XL_S
A+XL_S
B+XL_L
b2
c1
c2
c3
c4
c5
z1
z2
z3
z4
z5
z6
z8
c3
c5
c6
c7
c8
y5
intensity
mass
0e+00
1e+05
2e+05
3e+05
4e+05
5e+05
6e+05
500
1000
1500
2000
RDTNSKK+XL_S
A_b_ion
prec
A_y_ion
y1+
b2+
y2+
b5+
y5+
b6+
intensity
m/z
0
200
600
1000
200
300
400
500
600
700
800
RDTNSKK+XL_L
A_b_ion
prec
A_y_ion
y1+
b3++
b2+
y2+
b4+
b5+
y5+
b6+
intensity
m/z
0
1000
2000
200
400
600
800
LVPLKETIK+XL_S
LVPLKETIK+XL_L
